# The transverse abdominal muscle is excessively active during active straight leg raising in pregnancy-related posterior pelvic girdle pain: an observational study

**DOI:** 10.1186/s12891-017-1732-9

**Published:** 2017-08-25

**Authors:** Jan M. A. Mens, Annelies Pool-Goudzwaard

**Affiliations:** 1 Department of Rehabilitation Medicine & Physical Therapy, Erasmus University Medical Centre, PO Box 2040, 3000 CA Rotterdam, The Netherlands; 2 Vondellaan Clinic Vondellaan, 35Ac 2332AA Leiden, The Netherlands; 3 MOVE research Institute, Faculty of behavioural and movement sciences VU University Amsterdam, Amsterdam, The Netherlands

**Keywords:** Abdominal muscles, Low back/lumbar spine, Motor control/learning, Sacroiliac joint/pelvis, Ultrasound imaging

## Abstract

**Background:**

Many studies suggest that impairment of motor control is the mechanical component of the pathogenesis of painful disorders in the lumbo-sacral region; however, this theory is still unproven and the results and recommendations for intervention remain questionable. The need for a force to compress both innominate bones against the sacrum is the basis for treatment of pregnancy-related pelvic girdle pain (PGP). Therefore, it is advised to use a pelvic belt and do exercises to enhance contraction of the muscles which provide this compression. However, our clinical experience is that contraction of those muscles appears to be excessive in PGP. Therefore, in patients with long-lasting pregnancy-related posterior PGP, there is a need to investigate the contraction pattern of an important muscle that provides a compressive force, i.e. the transverse abdominal muscle (TrA), during a load transfer test, such as active straight leg raising (ASLR).

**Methods:**

TrA thickness was measured by means of ultrasound imaging at rest and during ASLR in 43 non-pregnant women with ongoing posterior PGP that started during a pregnancy or delivery, and in 39 women of the same age group who had delivered at least once and had no current PGP (healthy controls)*.*

**Results:**

In participants with PGP, the median TrA thickness increase with respect to rest during ipsilateral and contralateral ASLR was 31% (SD 46%) and 31% (SD 57%), respectively. In healthy controls, these values were 11% (SD 25%) and 13% (SD 22%), respectively.

**Conclusions:**

Significant excessive contraction of the TrA is present during ASLR in patients with long-lasting pregnancy-related posterior PGP. The present findings do not support the idea that contraction of the TrA is decreased in long-lasting pregnancy-related PGP. This implies that there is no rationale for the prescription of exercises to enhance contraction of TrA in patients with long-lasting pregnancy-related PGP.

**Electronic supplementary material:**

The online version of this article (doi:10.1186/s12891-017-1732-9) contains supplementary material, which is available to authorized users.

## Background

Low back pain (LBP) and pelvic girdle pain (PGP) or a combination of both, lumbopelvic pain (LPP) are common during pregnancy. Although a large proportion of women recover within one month after delivery, a substantial percentage (5–8.5%) has persisting complaints even up to 2 years after delivery [1]. Many studies suggest that impairment of motor control is the mechanical component of the pathogenesis of painful disorders in the lumbo-sacral region; [2] however, that theory is still unproven and the recommendations for intervention remain questionable.

The theory of compromised motor control to explain the development of LPP proposes that many activities create (small) movements in the lumbopelvic region, which subsequently induce strain on the ligaments and pain. For PGP this comprises the sacroiliac joints and the pubic symphysis, and the ligaments in the pelvic ring. Biomechanical and anatomical studies have shown that transversely-oriented muscles of the abdominal wall, especially the transverse abdominal muscle (TrA), in co-contraction with the pelvic floor, are the most suitable muscles to achieve compression of both innominate bones against the sacrum (‘force closure’ of the sacroiliac joints) and could, theoretically, reduce movement in the sacroiliac joints and the strain on the engaged ligaments [3–6]. The theory implies that the more efficiently individuals contract those muscles, the better they protect themselves against the strain on the ligaments in the pelvic ring, resulting in a lower risk to develop PGP and a greater chance to recover from it.

The theory would gain strength if the contraction of TrA during various tasks was shown to be smaller in patients with painful conditions than in healthy controls. However, the results of case-control studies in LPP are not consistent. A review from 2009 included three case-control studies that support the theory [7]. However, some studies published after that review (e.g. Beazell et al., 2011; Himes et al., 2012; Pinto et al., 2011) found no significant difference in TrA contraction between patients with LPP and controls [8–11]. Some limitations of previous studies include: heterogeneity of the study group with respect to gender, severity, duration and cause of LPP. Moreover, bias can be introduced if participants in the control group are not well defined (e.g. ‘convenient samples’, ‘colleagues’, etc.) due to differences in level of fitness (e.g. sports, work, absence from work, unemployment, fatigue, etc.). In addition, colleagues might have detailed knowledge of the objective of the study and this knowledge could influence ‘spontaneous, automatic recruitment’. Furthermore, in most studies, maximal voluntary muscle contraction was investigated instead of spontaneous, automatic recruitment of muscles during a well-defined task.

As a consequence, the recommendations for intervention are not unequivocal. For example, Teyhen et al. showed that contraction of the TrA during active straight leg raising (ASLR) is reduced in a well-defined subgroup of patients with LPP [12]. Therefore, they suggested that *‘patients with unilateral lumbopelvic pain who have a positive ASLR test may benefit from motor control exercises that specifically target activation of the deep abdominal musculature*’ [12]. O’Sullivan and Beales suggested that motor control impairments in long-lasting PGP show a large variation: ‘*non-specific’ PGP disorders are represented by a number of sub-groups with different underlying pain mechanisms rather than a single entity*.’ [13, 14] These authors recommend adapting the therapeutic intervention based on this sub-classification. Nevertheless, in physiotherapy for PGP, contraction of the TrA is emphasised, implying that the role of the muscle to compress both innominate bones against the sacrum is diminished. In contrast, our clinical experience is that contraction of those muscles appears to be excessive in PGP.

The contraction pattern of many muscles has been investigated in various subgroups of patients with LPP. In view of these earlier findings, it is important to establish the spontaneous activity of the muscles that provide a compressive force during a load transfer task in patients with long-lasting PGP. More specifically, during ASLR, the present study examines TrA thickness increase in a) women with long-lasting pregnancy-related posterior PGP compared with that in b) women from the same age group who had delivered at least once and have no current PGP (control group).

## Methods

A cross-sectional observational study was performed to address the research question. During ASLR, measurement of TrA thickness was performed by means of rehabilitative ultrasound imaging (RUSI). Increase of TrA thickness was regarded as contraction of the muscles, and decrease of thickness as a diminished contraction or relative relaxation. Thickness increase of the TrA during ASLR was computed with respect to rest and expressed in percentages of thickness at rest. Results of participants with PGP were compared with those of parous women without PGP. All participants were selected in a private orthopaedic clinic specialised in long-lasting pregnancy-related PGP.

### Study population

Inclusion criteria for pregnancy-related posterior PGP were: 1) pain in the area between the gluteal folds and the horizontal line through both iliac crests at the back (as indicated by the participant on a drawing), 2) the complaints were pregnancy related (defined as pain that started during pregnancy, or within 3 weeks after delivery, and ongoing since then), 3) the complaints were long-lasting, defined as pain persisting for a duration of >6 months, 4) a positive score on two tests for PGP, i.e. the ASLR test and the Posterior Pelvic Pain Provocation (P4) test (see below), and 5) less difficulty to perform the ASLR test after applying a pelvic belt.

Exclusion criteria (also for controls) were: 1) age < 18 or >55 years, 2) being pregnant or ≤6 months postpartum, 3) inability to fill in forms without the help of others, 4) indications for a specific cause of their pain (e.g. sciatica), 5) systemic disorders of the locomotor system (e.g. rheumatoid arthritis), and 6) bony or muscular abnormalities (traumatic, congenital or post-surgical).

Controls were recruited in the same clinic among individuals with minor ailments of the shoulder, elbow, wrist or feet. A control person was defined as a woman who had delivered at least once, without pain in the pelvic girdle region for at least 3 months, and had negative scores on both tests for PGP.

### Questionnaires and clinical examination

Using a questionnaire, information was collected on age, parity and general health. The questions on general health focused on the fulfilment of inclusion criteria, the possibility of exclusion criteria, and the severity and duration of complaints. Patients indicated the localisation of the pain on a drawing.

Severity of PGP was expressed as pain and as pain-related disability [15]. Pain was assessed on a numerical rating scale (ranging from 0 = no pain to 10 = the worst imaginable pain) by asking the patient to give the score of the average pain during the week before the examination [16]. Severity of pain-related disability was evaluated by means of the Quebec Back Pain Disability Scale. This questionnaire has good reliability and construct validity to measure disability [17, 18].

Two tests were used to diagnose PGP: the ASLR test and the P4 test [19]. The scores were given by the person on a 6-point Likert scale as described in an appraisal by Chang [20]. An ASLR score of 0 at both sides was defined as a negative test result, and all other scores as a positive test result [21].

The P4 test was performed in supine position with 90° hip flexion and 90° knee flexion [22]. In this position the investigator gave manual compression on the knee perpendicular to the examination table. The test was scored as positive if, at least at one side, pain was felt at the back of the pelvis at the tested side. Both the ASLR test and the P4 test have good reliability, and a high sensitivity and specificity for PGP [19, 20].

During a 4-year period (from 2007 to 08-1 until 2011–08-01), 43 consecutive patients with long-lasting posterior PGP and 39 controls without PGP were selected; these numbers of participants were arbitrarily chosen.

### Outcome measure

Outcome measure was the percentage change in TrA thickness during ASLR with respect to rest. Measurement of TrA thickness was performed by means of RUSI (2D mode) with a 7.5 MHz linear probe of 6 cm (Honda-Hs-2000). The examiner had 15 years of experience with RUSI. TrA thickness was measured at the right side of the body at the end of normal expiration in supine position. Measuring TrA thickness with the RUSI has high reliability and validity [7, 23]. Because many patients felt uncomfortable lying supine with the legs stretched, all measurements were performed with a small pillow placed under the bended knees. The ASLR test was performed as follows: first, the individual was asked to stretch the leg and subsequently to raise the extended leg some centimetres above the support. No instruction was given about breathing and/or tensing of the abdominal muscles. Participants had no view on the screen. RUSI was measured at the right side of the body, so ASLR right was indicated as ‘ipsilateral ASLR’ and left as ‘contralateral ASLR’.

**Fig. 1:**
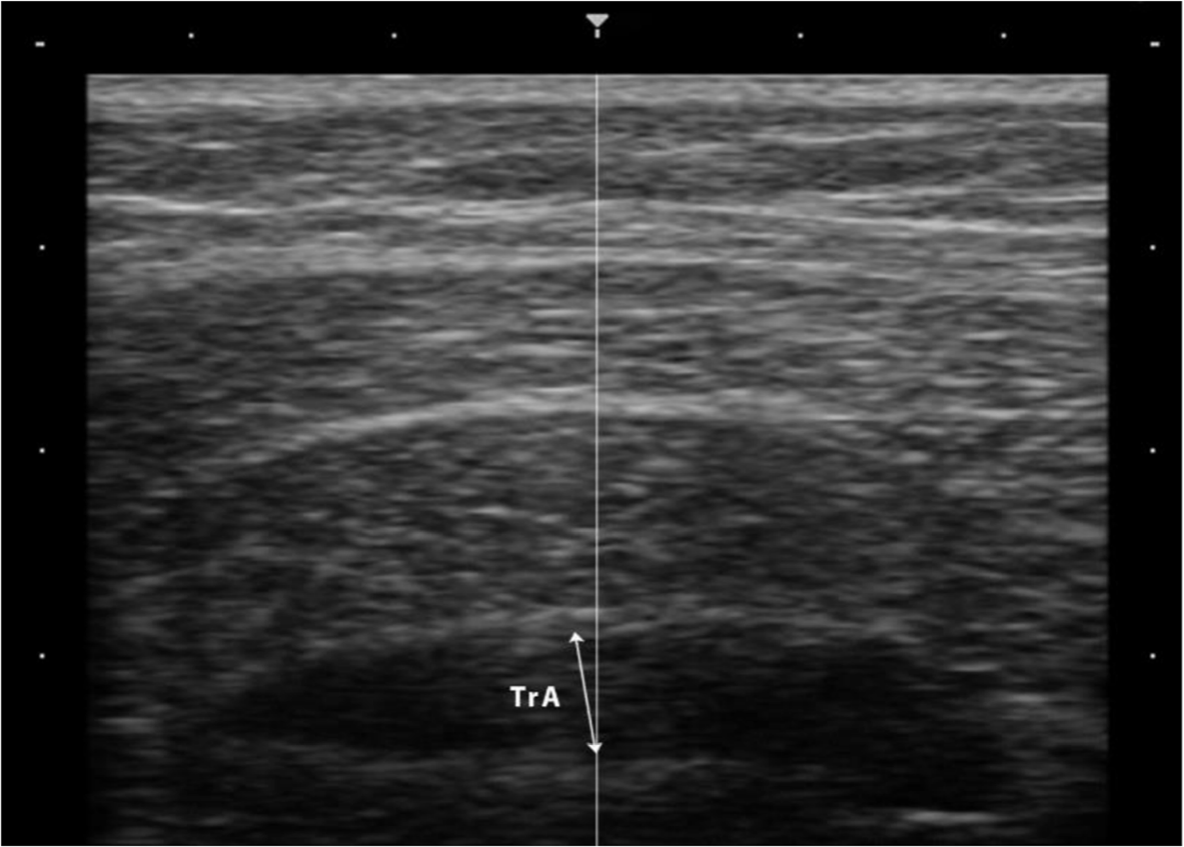
Transverse abdominal muscle (TrA) thickness was measured as the distance between both aponeuroses perpendicular to the direction of the muscle fibres

The probe was placed transversally halfway the right iliac crest and ribcage, and in such a way that the centre of the TrA was viewed in the centre of the image. Care was taken not to move the probe during measurements. Thickness was measured by the investigator immediately after production of the image (screen freeze) as the distance between both aponeuroses perpendicular to the direction of the muscle fibres (Fig. 1). TrA thickness was measured without any attempt to blind the investigator. Three series of TrA thickness measurement were performed. The series consisted of thickness measurement at rest, during ASLR contralateral, and during ASLR ipsilateral. After 2 min of rest, the next series started. Thus, the investigator was not blinded during the second and third series for the results of the previous measurements. The average value of three tests was used for the analysis. Outcome measure was the percentage change in TrA thickness during ASLR and was computed with the formula:

$$ \left(\left(\mathrm{thickness}\  \mathrm{during}\  \mathrm{ASLR}\hbox{--} \mathrm{thickness}\ \mathrm{at}\ \mathrm{rest}\right)/\mathrm{thickness}\ \mathrm{at}\ \mathrm{rest}\right)\times 100\%. $$

Intra-rater reliability was computed per condition separately in participants with and without PGP using intra-class correlation coefficient (ICC_2,1_).

### Statistical analysis

Normally distributed continuous variables are presented as mean and standard deviation (SD); non-normally distributed continuous variables as median and interquartile range. Differences between groups were analysed with an independent samples T-test in case of a normally distributed continuous variable and a Mann-Whitney test in case of a non-normal distribution or an ordinal variable. Differences were expressed as the 95% confidence interval (CI). A bootstrap method was used to compute the CI for non-normally distributed variables. The relation between TrA thickness increase during ASLR and possible confounders was checked per group and per side by means of Pearson’s rho; *p* < 0.05 was considered statistically significant. ICCs were interpreted according to Fleiss: ICC < 0.40 is a poor reliability; ICC ≥ 0.40 but ≤0.75 is a fair to good reliability; and ICC > 0.75 is an excellent reliability [24]. Analyses were performed with SPSS version 23. A significantly larger increase of TrA thickness with respect to healthy controls during ASLR was considered proof of ‘excessive activity’ of the TrA, and a significantly smaller increase was considered proof of ‘reduced activity’ of the TrA.

## Results

After applying the first three inclusion criteria, 77 women with pregnancy-related posterior pelvic pain of more than 6 months duration were selected. Of these, 31 could not be included due to a negative ASLR test (12 times) and/or a negative P4 test (18 times) and/or the absence of a positive effect of a pelvic belt on ASLR (15 times). In addition, two women were excluded because of ‘post-surgery bony abnormalities’ and one woman because her last delivery was less than 6 months ago. Thus, 43 participants were available for analysis. The characteristics of the 43 patients and 39 controls are presented in Table 1. On average, patients with PGP were about 4 years younger than controls, and duration since postpartum was also shorter by about 4 years. Of the 43 women with PGP, the pain started during pregnancy in 36 (84%) and within 3 weeks after delivery in 7 (16%). Posterior pelvic pain was felt at both sides in 31 patients (72%) and was unilateral in the remaining 28%.

**Table 1:** Characteristics of the participants with pelvic girdle pain (PGP) and controls without PGP

	PGP (*n* = 43)	Controls (*n* = 39)
Age in years, mean (SD)	36.7 (6.8)	41.1 (6.6)
Time since last delivery in years, median (IQR)	2.3 (3.0)	6.3 (6.4)
Number of vaginal deliveries, median (IQR)	2 (1)	2 (1)
Pain started during pregnancy, number (%)	36 (84)	n/a
Duration of complaints in years, median (IQR)	3.7 (5.1)	n/a
Bilateral pain, number (%)	31 (72)	n/a
Pain intensity (NRS), median (IQR)	6.0 (2.0)	0
Disability score (QBPDS), mean (SD)	54 (22)	0
ASLR score ipsilateral, median; (IQR)	2.5 (1.5)	0
ASLR score contralateral, median; (IQR)	2.5 (1.5)	0

*ASLR* active straight leg raising, *IQR* Interquartile range, *n/a* not applicable, *NRS* numeric rating scale, *QBPDS* Quebec Back Pain Disability Scale, Quebec Back Pain Disability Scale, *SD* standard deviation

Intra-rater reproducibility of TrA thickness measurements expressed as ICC_2,1_ for the three conditions ranged from 0.80–0.88 in patients with PGP and from 0.79–0.83 in controls; all these values could be labelled as ‘excellent’.

There was no significant difference in mean TrA thickness at rest between women with and without PGP (Table 2). In PGP, the median increase of the right TrA during ipsilateral ASLR and contralateral ASLR was 31% for both sides; this increase was significantly more than the 11% and 13%, respectively, of the controls (*p* < 0.001 and *p* = 0.005, respectively).

**Table 2:** Thickness of the right transverse abdominal muscle (TrA) in participants with pelvic girdle pain (PGP) and controls without PGP

	PGP (*n* = 43)	Controls (*n* = 39)	Group difference
TrA thickness at rest in mm, median (IQR)	3.1 (1.6)	3.1 (1.6)	0.17 (95% CI −0.25 to 0.54) *p* = 0.54
TrA thickness increase during ASLR ipsilateral in %, median (IQR)	31 (46)	11 (25)	25.7 (95% CI 14.9 to 41.2) *p* < 0.001
TrA thickness increase during ASLR contralateral in %, median (IQR)	31 (57)	13 (22)	23.6 (95% CI 10.4 to 37.5) *p =* 0.005

*ASLR* active straight leg raising, *CI* confidence interval, *IQR* Interquartile range

The relation between TrA thickness increase during ASLR and possible confounders was checked per group per side (ipsilateral/contralateral) for all the variables listed in Table 1. This revealed no significant correlations (analyses are shown in the Additional file 1: Table S1).

## Discussion

The main finding of the present study is that an excessive contraction of the TrA is present during ASLR in patients with long-lasting pregnancy-related posterior PGP. These results were independent of the duration or severity of their complaints, or their score on ASLR.

Strengths of the present study are: i) the homogeneity of the study group for gender and the cause of pain, ii) the size of the study group and the method used to study the spontaneous automatic muscle recruitment in response to a load transfer task, and iii) the use of a well-defined control group.

Although two earlier studies on motor control in pregnancy-related PGP also showed enlarged muscle activity during ASLR, TrA activity was not measured. One of these studies, investigating pregnant women with PGP, showed increased activity of the psoas major, external oblique (EO) and rectus abdominis during ASLR compared with controls [25]. In another study investigating long-lasting pregnancy-related PGP, indications were found for increased muscle tone of the pelvic floor, both at rest and during ASLR [26]. However, in two other studies a reduced muscle activity during ASLR was found. In a case-control study, Teyhen et al. showed that TrA and internal oblique (IO) were less active in patients with unilateral low back pain and a positive sacroiliac test [12]; however, in that study, the patients’ pain did not start during pregnancy or delivery (personal communication with Teyhen). Shadmehr et al. found a smaller recruitment of various muscles (EO, biceps femoris, gluteus maximus and erector spinae) during ASLR in women with sacroiliac pain compared with controls [27]; the participants of that study were not pregnant and at least 6 months postpartum. No information was available as to whether or not the pain started in relation to pregnancy (personal communication with Shadmehr).

It is a challenge to explain the difference between the excessive TrA contraction in the patients of the present study, and the reduced TrA contraction of the patients in the study of Teyhen et al. [11]. Nevertheless, one explanation could be the difference between the two study groups. In contrast with the present study, in the study of Teyhen et al. the pain of the participants did not start during pregnancy or delivery. In pregnancy-related PGP, enlarged mobility of the joints in the pelvic ring is well documented and this could be the key to the explanation (see below) [28].

It is also a challenge to explain why, in the present study, the enhanced contraction of the TrA in patients with PGP does not seem to help them to compress the pelvis sufficiently to perform the ASLR without difficulty. After all, in patients selected for this study the pelvic belt did help them to raise their leg.

We present two hypotheses. The first is related to a possible delay of TrA contraction during ASLR. A delay of TrA contraction is well-documented in electromyographic studies of patients with various painful disorders in the lumbopelvic region and in experimental pain [29–31]. It is feasible that, in case of enlarged mobility (and not in case of normal mobility), a small movement in the sacroiliac joints takes place during ASLR before contraction of the TrA. A radiographic study demonstrated the forward rotation of the innominate at the ipsilateral side during ASLR in pregnancy-related PGP [32]. It is possible that muscular bracing of the sacroiliac joints does not need a large TrA contraction, but rather a timely contraction (or a pelvic belt fastened before the ASLR) to prevent painful sacroiliac movements. It is also possible that pain caused by the small displacement and/or fear for more pain, induce not only a strong (but late) TrA contraction but also contraction of other muscles. A study in healthy volunteers demonstrated that excessive activity of IO, EO, rectus abdominis, biceps femoris and latissimus dorsi during ASLR could be induced by experimental pelvic pain [33]; unfortunately TrA activity was not measured in that study. Richardson et al. demonstrated how a large TrA contraction can be inefficient in stabilizing the pelvic ring [34]. They showed that the effect of TrA contraction on force closure is smaller when TrA contracts together with the IO and EO, than in selective TrA contraction; the authors introduced the term ‘bracing’ to describe the large, but inefficient, contraction of many trunk and hip muscles.

A second hypothesis is that TrA activity with RUSI is assessed halfway the iliac crest and ribcage (thus in the middle region of the TrA) and is not representative for TrA contraction of the pelvic part of the TrA. In healthy volunteers it has been shown that TrA activity can vary per region [35, 36]. Theoretically, excessive activity of the middle region of the TrA may exist in combination with insufficient activity of the pelvic part of the TrA. Contraction of the middle part of the TrA might support the lumbar spine and the lower part of the pelvic ring. Moreover, excessive contraction of the TrA in the middle region might have an adverse effect on the pelvic girdle due to increased intra-abdominal pressure (IAP). IAP increase during ASLR in patients with pelvic girdle pain is well documented [37]. The possible adverse effect of increased IAP on the pelvic ring has been demonstrated in a biomechanical study [38].

### Limitations

A limitation of the present study could be that TrA thickness increase is used as a measure of TrA contraction. It must be emphasised that TrA thickness increase is not one-to-one related to TrA contraction [39]. Although it is suggested that IAP and/or contraction of the IO might reduce TrA thickness due to compression, these two factors do not seem responsible for the greater increase in TrA thickness during ASLR in PGP. On the contrary, it is reported that IAP shows a greater rise during a positive ASLR than during a negative ASLR [37]. The same holds for contraction of the IO, i.e. De Groot et al. reported a larger IO contraction in patients with PGP than in controls [25].

Another limitation of the present study is the lack of blinding. TrA thickness was measured by the investigator who was aware whether the participant was a patient or a healthy control. Lack of blinding also limited the ICC assessment. Because the time period between the measurements was only 2 min, the investigator was able to remember the results of the previous measurement(s). Results need verification in an observer-blinded study.

Also, in the present study, because ASLR was performed by patients with severe, long-lasting pregnancy-related PGP, caution is required if extrapolating these results to other patient groups. Although no relation was found between the duration of complaints, patients with complaints lasting <6 months were lacking in the analysis. All patients in the present study had previously been treated with physiotherapy and (most likely) the majority had received instructions on how to improve TrA recruitment. It is possible that exercises to enhance contraction of the TrA in patients with PGP is justifiable for a considerable proportion of these patients and that the women in the present study represent a small selection of ‘non-responders’ that requires a different type of treatment.

## Conclusions

Significant excessive contraction of the TrA is present during ASLR in patients with long-lasting pregnancy-related posterior PGP. The results of this study do not support the idea that contraction of the TrA is decreased in long-lasting pregnancy-related PGP. This implies that there is no rationale for the prescription of exercises to enhance contraction of the TrA in patients with long-lasting pregnancy-related PGP.

## Additional file

Additional file 1: Table S1. Pearson’s rho correlations between TrA (transverse abdominal muscle) thickness increase and various characteristics of 43 participants with and 39 without pelvic girdle pain (PGP). No correlation was statistically significant. n/a, not applicable; ASLR, active straight leg raising. (DOCX 11 kb)

## Abbreviations

ASLR: Active Straight Leg Raising
CI: Confidence Interval
EO: external oblique muscle
IAP: Intra-abdominal pressure
ICC: Intra-class correlation coefficient
IO: Internal oblique muscle
LPP: Lumbopelvic pain
n/a: Not applicable
P4-test: Posterior Pelvic Pain Provocation test
PGP: Pelvic Girdle Pain
RUSI: Rehabilitative ultrasound imaging
SD: Standard deviation
TrA: Transverse abdominal muscle
