# Supplementary material for: The transverse abdominal muscle is excessively active during active straight leg raising in pregnancy-related posterior pelvic girdle pain: an observational study
Source: BMC Musculoskelet Disord. 2017 Aug 25;18:372. doi: 10.1186/s12891-017-1732-9 (PMC5574111; doi:10.1186/s12891-017-1732-9)
Supplement: Additional file 1: Table S1. — Pearson’s rho correlations between TrA (transverse abdominal muscle) thickness increase and various characteristics of 43 participants with and 39 without pelvic girdle pain (PGP). No correlation was statistically significant. n/a, not applicable; ASLR, active straight leg raising. (DOCX 11 kb) [file 12891_2017_1732_MOESM1_ESM.docx]

Supplementary table. Pearson’s rho correlations between TrA (transverse abdominal muscle) thickness increase and various characteristics of 43 participants with and 39 without pelvic girdle pain (PGP). No correlation was statistically significant. n/a, not applicable; ASLR, active straight leg raising.

|  | Participants with PGP | | Healthy controls | |
| --- | --- | --- | --- | --- |
|  | TrA thickness increase ipsilateral | TrA thickness increase contralateral | TrA thickness increase ipsilateral | TrA thickness increase contralateral |
| Age | -0.12 | -0.08 | -0.2 | -0.02 |
| Time since last delivery | 0.06 | 0.02 | -0.1 | 0.03 |
| Number of vaginal deliveries | 0.08 | 0.2 | -0.08 | 0.2 |
| Pain started during pregnancy | -0.1 | 0.0 | n/a | n/a |
| Duration of complaints | 0.04 | 0.01 | n/a | n/a |
| Bilateral pain | 0.04 | 0.004 | n/a | n/a |
| Pain intensity | -0.02 | -0.05 | n/a | n/a |
| Disability score (QBPDS) | 0.2 | 0.08 | n/a | n/a |
| ASLR score ipsilateral | 0.04 | -0.02 | n/a | n/a |
| ASLR score contralateral | 0.2 | 0.3 | n/a | n/a |
